# Striatofrontal Deafferentiation in MSA-P: Evaluation with [^18^F]FDG Brain PET

**DOI:** 10.1371/journal.pone.0169928

**Published:** 2017-01-13

**Authors:** Hae Won Kim, Minyoung Oh, Jungsu S. Oh, Seung Jun Oh, Sang Ju Lee, Sun Ju Chung, Jae Seung Kim

**Affiliations:** 1 Department of Nuclear Medicine, Keimyung University, School of Medicine, Daegu, Korea; 2 Department of Nuclear Medicine, Asan Medical Center, University of Ulsan College of Medicine, Seoul, Korea; 3 Department of Neurology, Asan Medical Center, University of Ulsan College of Medicine, Seoul, Korea; University of Manchester, UNITED KINGDOM

## Abstract

**Background:**

Although cognitive impairment is not a consistent feature of multiple system atrophy (MSA), increasing evidence suggests that cognitive impairment is common in MSA with predominant parkinsonism (MSA-P). It is assumed that the cognitive impairment in MSA-P is caused by the striatal dysfunction and disruption of striatofrontal connections. The aim of this study was to evaluate the relationship between regional glucose metabolism in the frontal cortex and striatum in patients with MSA-P using [^18^F]FDG brain PET.

**Methods:**

Twenty-nine patients with MSA-P and 28 healthy controls underwent [^18^F]FDG brain PET scan. The [^18^F]FDG brain PET images were semiquantitatively analyzed on the basis of a template in standard space. The regional glucose metabolism of the cerebral cortex and striatum were compared between MSA-P and healthy control groups. The correlations between age, symptom duration, H&Y stage, UPDRS III score, MMSE score, and glucose metabolism in the cerebellum and striatum to glucose metabolism in the frontal cortex were evaluated by multivariate analysis.

**Results:**

The glucose metabolism in the frontal cortex and striatum in MSA-P patients were significantly lower than those in healthy controls. Glucose metabolism in the striatum was the most powerful determinant of glucose metabolism in the frontal cortex in MSA-P. Only age and glucose metabolism in the cerebellum were independent variables affecting the glucose metabolism in the frontal cortex in healthy controls.

**Conclusion:**

The decrease in frontal glucose metabolism in MSA-P is related to the decrease in striatal glucose metabolism. This result provided evidence of striatofrontal deafferentiation in patients with MSA-P.

## Introduction

Multiple system atrophy (MSA) is a presumably sporadic neurodegenerative disorder characterized by central autonomic failure and additional degeneration of nigro-striatal or cerebellar circuits. Following current diagnostic criteria, MSA is classified into two subtypes according to the predominant symptoms: MSA with predominant parkinsonism (MSA-P) and MSA with cerebellar features (MSA-C) [1]. Although cognitive impairment in MSA is still defined as a non-supporting diagnostic feature by current consensus diagnostic criteria, increasing evidence suggests that cognitive impairment is common in both MSA subtypes [2–4]. Recent prospective neuropsychological studies estimated dementia prevalence rates in MSA of up to 31% [5, 6], and almost 50% of patients surviving more than 8 years are reported to be cognitively impaired [7]. Cognitive impairments in MSA occur across a wide spectrum from mild single domain deficits to impairments in multiple domains [7]. Frontal-executive dysfunction is the most common presentation, while memory and visuospatial functions also are impaired [7, 8]. Comparative studies of cognitive impairment in MSA-P and MSA-C showed that patients with MSA-P had more severe and more widespread cognitive dysfunctions than patients with MSA-C [9]. In accordance with the clinical features, [^18^F]FDG PET has shown a decrease in glucose metabolism not only in the putamen, pons, and cerebellum but also in the frontal and parieto-temporal cortices in patients with MSA [10, 11].

It is assumed that cognitive impairment in MSA is caused by striatal dysfunction and disruption of striatofrontal connections [4, 9]. Neuropathological findings support the concept that cognitive impairments in MSA originate from striatofrontal deafferentiation [2, 12, 13]. In Parkinson’s disease, many studies on cognitive impairment have revealed structural and functional abnormalities within the cortico-striato-thalamo-cortical circuits, known to be largely modulated by the dopaminergic system [14–16]. However, no study has yet demonstrated that the decreased cerebral cortical activity, which results in cognitive impairment, is correlated with striatal degeneration in patients with MSA. Although a histopathologic examination in the clinical field is limited, [^18^F]FDG PET can demonstrate the neuronal synaptic activity that correlates to striatonigral and cerebral cortical degeneration [11, 17, 18]. Alterations of functional connectivity in several brain diseases have been investigated by interregional correlation analysis using [^18^F]FDG PET [19, 20]. In this study, we evaluated the relationship between regional glucose metabolism in the frontal cortex and striatum in patients with MSA-P using [^18^F]FDG brain PET.

## Materials and Methods

### Subjects

Twenty-nine patients that underwent [^18^F]FDG brain PET scan and had clinically probable MSA-P were enrolled in this study. The clinical diagnoses were made by movement disorder specialists according to established criteria [1]. All patients were assessed by a neurologist specializing in movement disorders, and their medical records were made available. Motor disability related to parkinsonism was assessed in all patients in an off-drug state using Part III of the Unified Parkinson’s Disease Rating Scale (UPDRS III) and was classified according to the Hoehn & Yahr stage score (H&Y stage) immediately before the [^18^F]FDG PET. The cognitive test was performed within 1 month of the [^18^F]FDG PET study. General intellectual function was assessed using the mini-mental state examination (MMSE). Symptom duration was defined as the time interval from initial symptom onset to the time that patients underwent [^18^F]FDG PET. Twenty-eight healthy controls (18 men and 10 women; mean age 52.5 ± 7.0 years) were selected from the normal PET data pool maintained at the Asan Medical Center. Healthy controls were selected to have no neurological or psychological diseases. The institutional review board of the Asan Medical Center approved this study and all subjects signed a written informed consent form.

### PET Scan

The [^18^F]FDG PET was performed for all 29 patients with MSA-P and 28 healthy controls. All subjects fasted for at least 6 h before the scan, and the PET scans were performed in a quiet and dimly lit room. A transmission scan of 5 min using a ^68^Ge rotating pin source and an emission scan of 15 min were acquired 40 min after intravenous injection of 370 MBq [^18^F]FDG on the ECAT HR+ PET scanner (Siemens Medical Systems, Hoffman Estate, IL). The scanner imaged 63 planes with a slice thickness of 2.46 mm simultaneously for a longitudinal field of view of 15.5 cm. The transaxial field of view (FOV) was 22 cm and the matrix size was 128 × 128 (resulting voxel size: 1.72 × 1.72 × 2.46 mm^3^). The spatial resolution in the air (NEMA NU2-2001 test results) was 5 mm full width half maximum (FWHM). All emission images were reconstructed with ordered subset expectation maximization (OSEM) using 16 subsets and six iterations.

### Semiquantitative Analyses

Image processing was performed with SPM2 (Wellcome Department of Imaging Neuroscience, Institute of Neurology, University College London) within MATLAB 2013a (The MathWorks, Inc.) and MRIcro version 1.37 (Chris Rorden, Columbia, SC, USA, www.mricro.com). Semiquantitative analyses were based on volumes-of-interest (VOIs), as described previously [21]. All reconstructed PET images were spatially normalized to Montreal Neurology Institute (MNI) space (approximating Talairach space) using standard MNI PET templates. To analyze the striatum and the cerebellum, six VOIs for the bilateral ventral striatum, caudate nucleus, and putamen, and two VOIs for both cerebellar cortices were manually drawn on a canonical spatially-normalized T1 MRI image by an experienced nuclear medicine physician specializing in nuclear neurology. The VOI for the ventral striatum was defined on the basis of previously defined criteria [22], and the ventral striatum included the nucleus accumbens, the ventral caudate rostral to the anterior caudate, and the ventral putamen rostral to the anterior caudate. The outer boundaries of the striatal subregions were visually determined from the characteristic increased activity of the striatum, which readily distinguished these regions from extrastriatal structures. For analysis of the bilateral cerebral cortices, 20 regional VOIs were identified via the automated anatomic labeling (AAL) template, as previously described [23]. The regional VOIs for the cerebral cortices were the central region, lateral, medial, and orbital surfaces of the frontal lobe, lateral surface of temporal lobe, lateral and medial surfaces of parietal lobe, occipital lobe, limbic lobe, and insula. The VOI template in standard stereotactic space was automatically applied directly to the spatially normalized individual PET images to analyze glucose metabolism in the brain.

The activity concentration was calculated in each VOI of the [^18^F]FDG PET images. The occipital VOI was used as the non-specific reference tissue. The regional glucose metabolic ratio (MR) was defined as follows: MR = count of regional VOI/count of occipital VOI. The count was defined as counts per voxel in each VOI, more exactly, in [Bq/ml] units.

### Statistical Analyses

The age, MMSE score and MR of the bilateral striatum, cerebellum, and cerebral cortices were compared between the healthy control and MSA-P groups using student’s t tests. The sex difference between each group was evaluated with the chi-square test. The relationships between MR of the ipsilateral frontal lobe, and age, symptom duration, H&Y stage, UPDRS III score, MMSE score, MR of the contralateral cerebellum, and MR of the ipsilateral striatum were analyzed by Pearson correlations and simple linear regression analyses. The relationships between MR of the ipsilateral frontal lobe and MR of the ipsilateral striatum were assessed by multiple linear regression analyses to adjust for other possible covariables, including age and MR of the contralateral cerebellum. The *p-*values were corrected for multiple comparisons using a false discovery rate correction. A *p-*value of less than 0.05 was considered to indicate statistical significance, and the data for the study variables were expressed as mean ± SD.

## Results

### Comparison of Striatal and Cortical Metabolism

Table 1 summarizes clinical characteristics of the MSA-P patients and healthy controls. The MMSE scores of the patients in the MSA-P group were significantly lower than those of the healthy control group (*p* = 0.31) There were no significant differences in age and sex between healthy control and MSA-P groups.

**Table 1:** clinical characteristics of subjects.

Characteristics	Control (n = 28)	MSA-P (n = 29)
Age (y)	53 ± 7	57 ± 15
Sex (M/F)	18 / 10	12 / 17
Symptom duration (y)	NA*	3.3 ± 2.2
H&Y stage	NA	3.2 ± 1.4
UPDRS III score	NA	33.8 ± 15.4
MMSE score	28.0 ± 1.5	26.5 ± 3.1

*NA = not applicable; Values are reported as mean ± SD, otherwise indicated.

In comparisons of the striatum and cerebellar metabolism, MRs of the right ventral striatum, bilateral caudate nucleus, putamen, and cerebellum in MSA-P patients were significantly lower than those in healthy controls. In comparisons of cerebral cortical glucose metabolism, MRs of the lateral surface of the right frontal lobe, the orbital surfaces of the bilateral frontal lobes, and the right insula were significantly lower in MSA-P patients than in healthy controls (Table 2).

**Table 2:** Comparison of metabolic ratios between healthy control and MSA-P.

VOI	Side	Control (n = 28	MSA-P (n = 29)	Corrected *p*
**Striatum**				
Ventral striatum	Left	1.11 ± 0.05	1.01 ± 0.08	<0.001*
	Right	1.07 ± 0.06	1.03 ± 0.09	0.054
Caudate nucleus	Left	1.00 ± 0.07	0.80 ± 0.12	<0.001*
	Right	1.12 ± 0.05	1.02 ± 0.12	<0.001*
Putamen	Left	1.16 ± 0.05	0.93 ± 0.14	<0.001*
	Right	1.19 ± 0.05	1.02 ± 0.12	<0.001*
**Cerebellum**	Left	0.89 ± 0.06	0.77 ± 0.15	0.001*
	Right	0.93 ± 0.07	0.81 ± 0.06	0.004*
**Cerebral cortex**				
Central region	Left	1.00 ± 0.03	1.01 ± 0.06	0.609
	Right	1.00 ± 0.04	1.00 ± 0.06	0.816
Frontal lobe, lateral surface	Left	1.02 ± 0.05	1.00 ± 0.05	0.271
	Right	1.05 ± 0.05	0.99 ± 0.06	<0.001*
Frontal lobe, medial surface	Left	0.97 ± 0.04	0.96 ± 0.06	0.609
	Right	0.98 ± 0.04	0.98 ± 0.06	0.609
Frontal lobe, orbital surface	Left	0.98 ± 0.04	0.94 ± 0.05	0.010*
	Right	1.03 ± 0.05	0.97 ± 0.07	0.001*
Temporal lobe, lateral surface	Left	1.02 ± 0.03	1.00 ± 0.06	0.159
	Right	1.03 ± 0.04	1.00 ± 0.07	0.058
Parietal lobe, lateral surface	Left	1.02 ± 0.03	0.99 ± 0.05	0.059
	Right	1.01 ± 0.04	0.99 ± 0.05	0.159
Parietal lobe, medial surface	Left	1.06 ± 0.04	1.06 ± 0.07	0.944
	Right	1.03 ± 0.04	1.06 ± 0.06	0.148
Limbic lobe	Left	0.87 ± 0.04	0.84 ± 0.06	0.058
	Right	0.86 ± 0.04	0.84 ± 0.05	0.159
Insula	Left	0.94 ± 0.04	0.92 ± 0.07	0.151
	Right	0.96 ± 0.05	0.92 ± 0.07	0.047*

*Statistically significant results.

### Relationship between Striatal and Frontal Metabolism

**Fig 1:**
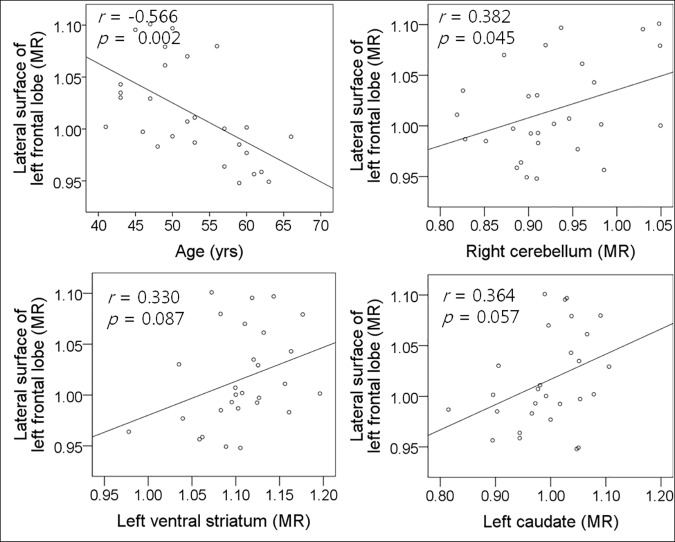
Relationships between left frontal glucose metabolism, and age, cerebellar and striatal glucose metabolism in healthy control.

**Fig 2:**
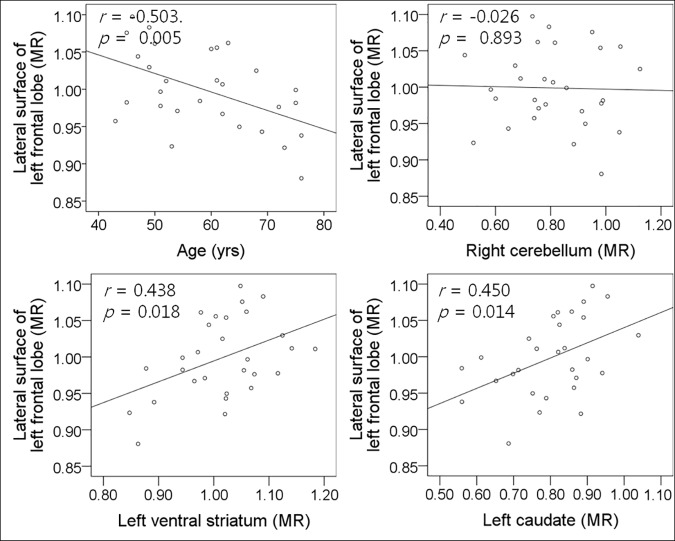
Relationships between left frontal glucose metabolism, and age, cerebellar and striatal glucose metabolism in MSA-P.

To identify the correlations between MR of the ipsilateral frontal lobe and age, symptom duration, H&Y stage, UPDRS III score, MR of contralateral cerebellum, or MR of ipsilateral striatum, Pearson correlation analyses were performed in healthy control and MSA-P groups. In healthy controls, MR of the right cerebellum correlated positively to MR of the lateral and orbital surfaces of the left frontal lobe (Fig 1). There were moderate negative correlations between age and MR of the lateral, medial, and orbital surfaces of the left frontal lobe. There was no significant correlation between MRs of the left striatum and left frontal lobe except between MRs of the left ventral striatum and orbital surface of the left frontal lobe (S1 Table). In MSA-P patients, age and MRs of the left ventral striatum and left caudate nucleus correlated to MR of the lateral and orbital surfaces of the left frontal lobe (Fig 2). The symptom duration, H&Y stage, UPDRS III score, MMSE score, and MRs of the left putamen and right cerebellum did not correlate to MRs of the left frontal lobe. The relationships between MR of the right frontal lobe and age, symptom duration, H&Y stage, UPDRS III score, MMSE score, and MRs of the left cerebellum and right striatum were similar to those of the left frontal lobe (S2 Table).

Multiple linear regression analyses showed that age and MR of the right cerebellum were significant independent variables to MR of the lateral and orbital surfaces of the left frontal lobe, and only age was an independent variable to MR of medial surfaces of the left frontal lobe in healthy control (Table 3). In MSA-P patients, MR of the left ventral striatum was the most powerful determinant of MR of the lateral and orbital surfaces of the left frontal lobe (Table 4). In addition, age was an independent variable to MR of the lateral surface of the left frontal lobe, and MR of the right cerebellum was an independent variable to MR of orbital surface of the left frontal lobe. To MR of medial surface of the left frontal lobe, only age was an independent variable. To MRs of right frontal lobe, multiple linear regression analysis showed the similar results with left frontal lobe in healthy control and MSA-P groups.

**Table 3:** Result of linear regression analysis for determinants of frontal glucose metabolism in healthy control.

Variables	Univariate model		Multivariate model		
	Adjusted *R*^2^	*p*	Adjusted *R*^2^	Standardized beta	*p*
Left frontal lobe, lateral surface					
Age	0.259	0.006*	0.401	-0.426	0.010*
Left ventral striatum	0.123	0.067			
Left caudate nucleus	0.194	0.019*			
Left putamen	0.170	0.029*			
Right cerebellum	0.271	0.005*		0.440	0.008*
Left frontal lobe, medial surface					
Age	0.172	0.028*	0.172	-0.415	0.028*
Left ventral striatum	0.040	0.313			
Left caudate nucleus	0.038	0.318			
Left putamen	0.031	0.368			
Right cerebellum	0.000	0.987			
Left frontal lobe, orbital surface					
Age	0.203	0.016*	0.528	-0.341	0.022*
Left ventral striatum	0.062	0.201			
Left caudate nucleus	0.168	0.030*			
Left putamen	0.153	0.040*			
Right cerebellum	0.416	0.001*		0.581	<0.001*
Right frontal lobe, lateral surface					
Age	0.320	0.002*	0.388	-0.538	0.002*
Right ventral striatum	0.109	0.087			
Right caudate nucleus	0.133	0.057			
Right putamen	0.054	0.236			
Left cerebellum	0.146	0.045*		0.338	0.035*
Right frontal lobe, medial surface					
Age	0.258	0.006*	0.258	-0.507	0.006*
Right ventral striatum	0.029	0.389			
Right caudate nucleus	0.060	0.208			
Right putamen	0.047	0.269			
Left cerebellum	0.000	0.978			
Right frontal lobe, orbital surface					
Age	0.303	0.002*	0.635	-0.433	0.001*
Right ventral striatum	0.221	0.012*		0.299	0.021*
Right caudate nucleus	0.133	0.056			
Right putamen	0.046	0.273			
Left cerebellum	0.339	0.001*		0.510	<0.001*

*Statistically significant results.

**Table 4:** Result of linear regression analysis for determinants of frontal glucose metabolism in MSA-P.

Variables	Univariate model		Multivariate model		
	Adjusted *R*^2^	*p*	Adjusted *R*^2^	Standardized beta	*p*
Left frontal lobe, lateral surface					
Age	0.149	0.022*	0.502	-0.322	0.025*
Left ventral striatum	0.416	< 0.001*		0.607	<0.001*
Left caudate nucleus	0.092	0.111			
Left putamen	0.122	0.063			
Right cerebellum	0.040	0.298			
Left frontal lobe, medial surface					
Age	0.192	0.010*	0.192	-0.470	0.010*
Left ventral striatum	0.058	0.209			
Left caudate nucleus	0.077	0.146			
Left putamen	0.103	0.090			
Right cerebellum	0.010	0.601			
Left frontal lobe, orbital surface					
Age	0.007	0.659			
Left ventral striatum	0.565	< 0.001*	0.654	0.717	<0.001*
Left caudate nucleus	0.086	0.123			
Left putamen	0.132	0.053			
Right cerebellum	0.144	0.024*		0.317	0.009*
Right frontal lobe, lateral surface					
Age	0.240	0.007*	0.306	-0.414	0.016*
Right ventral striatum	0.162	0.018*		0.348	0.040*
Right caudate nucleus	0.173	0.014*			
Right putamen	0.069	0.168			
Left cerebellum	0.001	0.894			
Right frontal lobe, medial surface					
Age	0.148	0.022*	0.148	-0.423	0.022*
Right ventral striatum	0.075	0.150			
Right caudate nucleus	0.123	0.062			
Right putamen	0.066	0.178			
Left cerebellum	0.006	0.702			
Right frontal lobe, orbital surface					
Age	0.184	0.012*	0.368	-0.362	0.027*
Right ventral striatum	0.262	0.003*		0.459	0.006*
Right caudate nucleus	0.207	0.008*			
Right putamen	0.065	0.182			
Left cerebellum	0.019	0.478			

* Statistically significant results.

## Discussion

This study demonstrated a positive correlation between frontal cortical activity and striatal activity in MSA-P. Decreased glucose metabolism in the bilateral frontal cortex and striatum was noted in MSA-P patients compared with healthy controls, and the glucose metabolism of the frontal cortex was positively correlated with the glucose metabolism of the striatum in MSA-P. Multivariate analysis showed that glucose metabolism of the ventral striatum was the most powerful determinant of glucose metabolism of the frontal cortex in MSA-P, while the determinant in healthy controls was not glucose metabolism of the striatum, but age and glucose metabolism of the cerebellum. This is the first study to demonstrate the striatofrontal deafferentiation in patients with MSA-P using [^18^F]FDG PET scan.

Recent reports suggest that cognitive impairment in MSA is more frequent than previously recognized [5–7]. Mild to moderate deficits in executive functions and impairments in attention and phonemic fluency were frequently observed in patients with MSA [5, 7]. Comparative study on subtypes of MSA revealed that patients with MSA-P tended toward a wide and severe impairment in cognitive function compared with patients with MSA-C [9]. Many imaging studies also showed cortical degeneration in MSA. MRI studies indicated a characteristic pattern of prefrontal, frontal, temporal, and parietal cortical atrophy in MSA-P [13, 24]. The cortical atrophy is supported by decreased glucose metabolism on [^18^F]FDG PET in prefrontal, frontal, temporal, and parietal regions in neuropathologically confirmed MSA-P [25]. Significant correlations between decreased glucose metabolism or decreased perfusion in the frontal cortex and executive function have been reported in MSA [9, 11]. We found decreased glucose metabolism in the frontal cortex, which is in agreement with results of previous studies.

Many imaging and neuropathological studies indicated that cognitive impairments in MSA originate from striatofrontal deafferentiation, with additional contributions from intrinsic cortical degeneration and cerebellar pathology. Paviour et al. [26] reported a correlation between striatal atrophy and impairment in different cognitive domains as well as global cognition in patients with MSA. An MRI study with voxel-based morphometry revealed that volume change in the caudate nucleus correlated significantly with cognitive test scores [27]. Despite the lack of detailed neuropsychological studies in patients with pathologically proven MSA, the similarity of widespread subcortical pathology in other degenerative basal ganglia disorders indirectly suggests that the disruption of subcortico-cortical pathways is likely to mediate some of the cognitive impairment in MSA [14, 16, 28]. On the other hand, post-mortem evidence of cortical degeneration supports additional primary cortical involvement in the cognitive deficits found in MSA [29–31]. Neuronal loss, astrogliosis, and loss of myelinated fibers in deeper cortical layers of the frontal lobes, abundant glial cytoplasmic inclusions found in deep cortical gray matter and the white matter of frontal and parietal lobes [29, 30], and the vacuolation of glial cells in the frontal cortex [31] point toward prominent frontal degeneration in MSA. Pathologic studies have revealed that the putamen are is more severely involved than the caudate nucleus and the ventral striatum in MSA [32, 33]. However, we found that glucose metabolism in the ventral striatum was the most powerful determinant of glucose metabolism in the frontal lobe and that glucose metabolism in the putamen had a weak correlation with glucose metabolism in the frontal lobe. This finding is in agreement with probabilistic diffusion tractography studies with connectivity analysis showing that the ventral striatum is functionally connected to the frontal cortex, and that the putamen is connected to the supplementary motor and primary motor areas. Although our results cannot be used to exclude the possibility that the frontal cortex is directly affected by α-synuclein pathology in MSA, they support the idea that secondary disruption of striatocortical connectivity may, at least partially, contribute to decreased neuronal activity in the frontal lobe in MSA.

Cerebral glucose metabolism correlates negatively with age [34] and correlates positively with contralateral cerebellar glucose metabolism [35]. In accordance with previous studies, the present study showed that not only striatal glucose metabolism but also age and cerebellar glucose metabolism are independent variables to frontal glucose metabolism in MSA-P. In healthy controls, only age and cerebellar glucose metabolism are independent variables to frontal glucose metabolism. Although there was also striatofrontal connectivity in healthy controls, a weak correlation between the striatal and frontal glucose metabolism would result from the many variable factors other than striatal activity that influence frontal cortical activity in healthy brain [18–20]. Decreased glucose metabolism in the frontal cortex with age does not appear to be mediated by amyloid pathology, as decreased glucose metabolism appears to begin before the age of 60 years, before there is appreciable accumulation of β-amyloid in the cerebral cortex [36]. Cortical metabolic changes observed in normal aging support the developmental theory, in which age-related brain changes follow phylogenetic and ontogenetic axes [37]. The topographic pattern of decreased glucose metabolism in normal aging matches in a reverse sense the metabolic functional changes observed in developing human brain. A correlation between ipsilateral frontal and contralateral cerebellar glucose metabolism is attributable to modulation by the frontal cortical activity in the contralateral cerebellum, because the corticopontine-pontocerebellar system is the largest system linking the ipsilateral cerebral cortex and contralateral cerebellum [38].

The reference tissue assignment is important for semiquantitative assessment of glucose metabolism, because the variability of activity concentration in the reference tissue could greatly affect the study results. In most studies using [^18^F]FDG brain PET, the whole brain has been used as the reference, and the image data were proportionally scaled to the cerebral global mean count [10, 14, 39]. However, global count normalization could underestimate the regional cerebral glucose metabolism in patients with MSA, because several studies with MSA have shown decreased glucose metabolism in most parts of the brain, including the striatum, cerebellum, brainstem, and cerebral cortex [26, 39, 40]. Therefore, cerebral regions that have preserved glucose metabolism should be assigned as the reference for more accurate semiquantitative analysis in patients with MSA. Lyoo et al. [11] and Teune et al. [39] reported that SPM analysis of FDG PET showed decreased glucose metabolism in the putamen and cerebellar hemisphere, as well as decreased glucose metabolism in the parietotemporal cortex, prefrontal cortex, and motor cortex in patients with MSA, but no significant change in the occipital cortex. Pathologic reports also revealed numerous glial cytoplasmic inclusions in the frontal cortex and white matter and neuronal loss in the frontal and temporal cortices, but very few glial cytoplasmic inclusions in the occipital cortex [30, 41]. The present study thus used the occipital cortex as the non-specific reference tissue, and MR could be calculated consistently.

Although cognitive impairment is relatively frequent in MSA-P, it involves primarily frontal-executive functions. General cognitive decline as assessed by MMSE is uncommon, but frontal lobe-related functions as measured by the frontal assessment battery and Raven's colored progressive matrices were frequently impaired in MSA-P [8, 9]. In the present study, cognitive function as assessed by MMSE was not correlated with frontal glucose metabolism. This may be due to the limitations of this retrospective study, as the MMSE is not an ideal cognitive assessment method for patients with MSA and we did not perform frontal executive function tests. Although we found secondary disruption of striatofrontal connectivity in MSA-P, we were unable to determine the effects of striatofrontal deafferentation on cognition. A prospective study with frontal executive function tests could reveal significant correlations between frontal glucose metabolism and executive function, and between striatal metabolism and executive function in patients with MSA-P.

## Conclusions

The present study demonstrated that the decreased frontal glucose metabolism in MSA-P is related to the decreased striatal glucose metabolism. This result provides evidence of striatofrontal deafferentiation in patients with MSA-P. Further studies with frontal executive function tests will be required to ascertain that cognitive impairments in MSA-P originate from striatofrontal deafferentiation as a result of subcortical pathology.

## Supporting Information

S1 Data Data underlying the findings. (XLSX)

S1 Table Relationships between left frontal glucose metabolism, and clinical characteristics, striatal and cerebellar glucose metabolism. (DOCX)

S2 Table Relationships between right frontal glucose metabolism, and clinical characteristics, striatal and cerebellar glucose metabolism. (DOCX)
