# Supplementary material for: Striatofrontal Deafferentiation in MSA-P: Evaluation with [18F]FDG Brain PET
Source: PLoS One. 2017 Jan 13;12(1):e0169928. doi: 10.1371/journal.pone.0169928 (PMC5234778; doi:10.1371/journal.pone.0169928)
Supplement: S1 Table — (DOCX) [file pone.0169928.s002.docx]

**S1 Table.** Relationships between left frontal glucose metabolism, and clinical characteristics, striatal and cerebellar glucose metabolism

| Variables^*^ | Control | | MSA-P | |
| --- | --- | --- | --- | --- |
|  | *r* | *p* | *r* | *p* |
| Frontal lobe, lateral surface |  |  |  |  |
| Age | -0.566 | 0.002^‡^ | -0.503 | 0.005^‡^ |
| Symptom duration | NA |  | 0.204 | 0.289 |
| H&Y stage | NA |  | -0.041 | 0.875 |
| UPDRS III score | NA |  | -0.051 | 0.852 |
| MMSE score | NA |  | 0.243 | 0.383 |
| Ventral striatum | 0.330 | 0.087 | 0.438 | 0.018^‡^ |
| Caudate nucleus | 0.364 | 0.057 | 0.450 | 0.014^‡^ |
| Putamen | 0.232 | 0.236 | 0.263 | 0.168 |
| Cerebellum^†^ | 0.382 | 0.045^‡^ | -0.026 | 0.893 |
| Frontal lobe, medial surface |  |  |  |  |
| Age | -0.507 | 0.006^‡^ | -0.305 | 0.108 |
| Symptom duration | NA |  | -0.014 | 0.941 |
| H&Y stage | NA |  | -0.082 | 0.753 |
| UPDRS III score | NA |  | -0.060 | 0.825 |
| MMSE score | NA |  | 0.077 | 0.785 |
| Ventral striatum | 0.169 | 0.389 | 0.274 | 0.150 |
| Caudate nucleus | 0.245 | 0.208 | 0.351 | 0.062 |
| Putamen | 0.216 | 0.269 | 0.257 | 0.178 |
| Cerebellum^†^ | -0.005 | 0.978 | -0.074 | 0.702 |
| Frontal lobe, orbital surface |  |  |  |  |
| Age | -0.550 | 0.002^‡^ | -0.376 | 0.044^‡^ |
| Symptom duration | NA |  | 0.112 | 0.562 |
| H&Y stage | NA |  | -0.202 | 0.438 |
| UPDRS III score | NA |  | -0.095 | 0.725 |
| MMSE score | NA |  | 0.025 | 0.930 |
| Ventral striatum | 0.470 | 0.012^‡^ | 0.537 | 0.003^‡^ |
| Caudate nucleus | 0.365 | 0.056 | 0.486 | 0.008^‡^ |
| Putamen | 0.215 | 0.273 | 0.255 | 0.182 |
| Cerebellum^†^ | 0.582 | 0.001^‡^ | 0.137 | 0.479 |

^*^Left side except the cerebellum; ^†^Right cerebellum; ^‡^Statistically significant results
