# Supplementary material for: Striatofrontal Deafferentiation in MSA-P: Evaluation with [18F]FDG Brain PET
Source: PLoS One. 2017 Jan 13;12(1):e0169928. doi: 10.1371/journal.pone.0169928 (PMC5234778; doi:10.1371/journal.pone.0169928)
Supplement: S2 Table — (DOCX) [file pone.0169928.s003.docx]

**S2 Table.** Relationships between right frontal glucose metabolism, and clinical characteristics, striatal and cerebellar glucose metabolism

| Variables^*^ | Control | | MSA-P | |
| --- | --- | --- | --- | --- |
|  | r | *p* | r | *p* |
| Frontal lobe, lateral surface |  |  |  |  |
| Age | -0.509 | 0.006^‡^ | -0.376 | 0.044^‡^ |
| Symptom duration |  |  | 0.022 | 0.910 |
| H&Y stage |  |  | -0.043 | 0.870 |
| UPDRS III score |  |  | -0.081 | 0.766 |
| MMSE score |  |  | 0.039 | 0.891 |
| Ventral striatum | 0.351 | 0.067 | 0.661 | <0.001^‡^ |
| Caudate nucleus | 0.440 | 0.019^‡^ | 0.303 | 0.111 |
| Putamen | 0.412 | 0.029^‡^ | 0.350 | 0.063 |
| Cerebellum^†^ | 0.520 | 0.005^‡^ | 0.200 | 0.298 |
| Frontal lobe, medial surface |  |  |  |  |
| Age | -0.415 | 0.028^‡^ | -0.334 | 0.077 |
| Symptom duration |  |  | -0.049 | 0.802 |
| H&Y stage |  |  | 0.201 | 0.440 |
| UPDRS III score |  |  | 0.010 | 0.969 |
| MMSE score |  |  | 0.216 | 0.439 |
| Ventral striatum | 0.198 | 0.313 | 0.240 | 0.209 |
| Caudate nucleus | 0.196 | 0.318 | 0.277 | 0.146 |
| Putamen | 0.177 | 0.368 | 0.320 | 0.091 |
| Cerebellum^†^ | 0.004 | 0.986 | -0.101 | 0.601 |
| Frontal lobe, orbital surface |  |  |  |  |
| Age | -0.451 | 0.016^‡^ | -0.086 | 0.659 |
| Symptom duration |  |  | -0.123 | 0.524 |
| H&Y stage |  |  | 0.003 | 0.992 |
| UPDRS III score |  |  | -0.053 | 0.845 |
| MMSE score |  |  | -0.178 | 0.525 |
| Ventral striatum | 0.249 | 0.201 | 0.762 | <0.001^‡^ |
| Caudate nucleus | 0.410 | 0.030^‡^ | 0.293 | 0.123 |
| Putamen | 0.391 | 0.040^‡^ | 0.363 | 0.053 |
| Cerebellum^†^ | 0.645 | <0.001^‡^ | 0.418 | 0.024^‡^ |

^*^Right side except the cerebellum; ^†^Left cerebellum; ^‡^Statistically significant results
